# Pretreatment of Mice with 830 nm Light Enhances Endurance During Acute Exercise

**DOI:** 10.3390/muscles4040048

**Published:** 2025-10-23

**Authors:** Nashwa Cheema, Namrata Ghag, Linh Pham, Emma Wise, Christiane Fuchs, Rox Anderson, Joshua Tam

**Affiliations:** 1 Wellman Center for Photomedicine, Massachusetts General Hospital, Boston, MA 02114, USA; 2 Department of Dermatology, Harvard Medical School, Boston, MA 02114, USA; 3 Harvard-MIT Health Sciences and Technology, Massachusetts Institute of Technology, Cambridge, MA 02139, USA

**Keywords:** photobiomodulation, near-infrared light, low-level light therapy, mouse, skeletal muscle, exercise, fatigue, mitochondria

## Abstract

Light therapy has been shown to produce several beneficial physiological effects in a wide range of tissues. The musculoskeletal system can be irradiated with deeply penetrating wavelengths in near infrared (NIR) regions. Photobiomodulation therapy (PBMT) reduces pain and inflammation and enhances physical performance. However, the mechanism(s) of cellular responses to PBMT in muscle is not clearly understood. Therefore, the goal of this study is to improve our understanding of the mechanism(s) of action of PBMT effects in exercised and sedentary muscle. In sedentary mice, PBMT using a wavelength of 830 nm increased the gene expression for muscle tissue development, including cFos, which is critical for activating interstitial and satellite cells that repair muscle. Immunostaining for cFOS expression confirmed an increase in the number of activated cells in PBMT-treated muscle. We observed that PBMT-treated mice showed increased performance on the treadmill, reduced muscle fiber damage, and altered mitochondrial structure. RNA sequencing from fatigued TA tissue suggested that PBMT treatment increased the gene expression of tissue regeneration and remodeling, suggesting tissue adaptation and muscle repair after exercise with PBMT. In conclusion, our study suggests that the 830 nm wavelength may have altered the muscle by activating regenerative genes that protect the tissue from exercise-induced cellular stress.

## 1. Introduction

Photobiomodulation (PBM) broadly refers to physiologic changes caused by photochemical reactions induced by light absorption. The mechanism of PBM is mediated through chromophores present in the cells that absorb energy from light and become activated. PBM modulates the biological response of the cells through higher activity of the election transport chain via the chromophore cytochrome c oxidase (COX) in the mitochondria. COX absorbs light in the wavelengths of 600–900 nm and increases ATP levels within the cell [1]. The use of photobiomodulation therapy (PBMT) has become a common practice in sports medicine; it is used to reduce inflammation, muscular injuries, and fatigue and to prevent performance decline [2]. However, its biological mechanisms are not clearly understood.

PBMT treatment has been shown to enhance muscle regeneration and prevent fatigue in rodent models of exercise [3], muscle wasting [4], and muscle disuse [5]. In in vivo models, increased inflammation mainly contributes to the pathophysiology of muscle wasting. PBMT has been shown to decrease inflammation, improve anti-oxidant signaling, and prevent muscle deterioration. In isolated muscle cells, proliferation was increased, suggesting an effect on muscle stem cells [4]. The activation of satellite cells, i.e., specialized muscle stem cells, with PBMT has been previously observed in rodents with muscle disuse [5]. However, the effect of PBMT on specific pathways that activate muscle recovery during exercise is unknown.

Exercise consists of either resistance training to improve strength or training on the treadmill to improve physical endurance. In humans, the effect of PBMT has been observed to be beneficial for both training types [6]. Most studies in rodents have revealed similar PBMT effects to those seen in humans, although they have been focused on resistance training [7,8], with limited studies on physical endurance [9]. Hence, this study is focused on endurance performance in healthy mice to fully understand the impact of PBMT application on muscle.

## 2. Results

### 2.1. PBMT Treatment Can Induce Upregulation of Regenerative Genes and Differentiation in Sedentary Muscle

**Figure 1:**
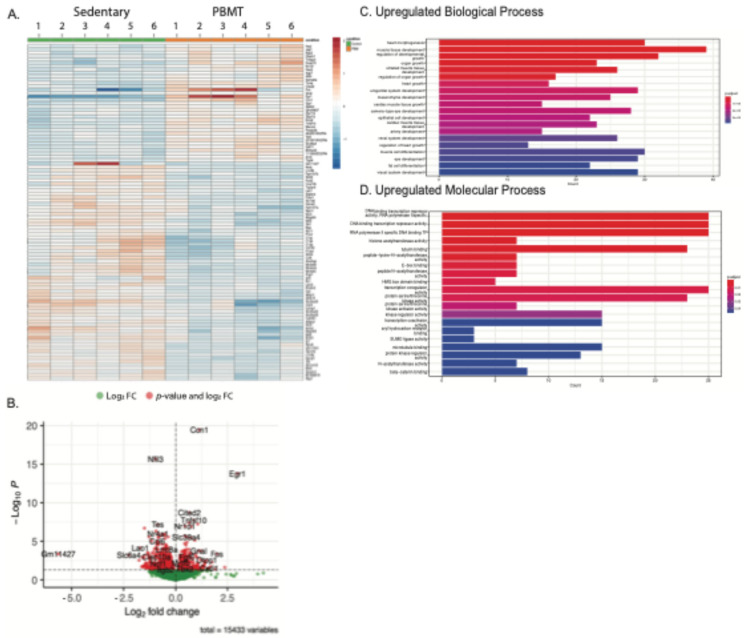
Upregulation of developmentally specific genes in PBMT sedentary TA muscle. (**A**) After 24 h, sedentary PBMT-treated and control mice tissue showed significant increases in genes expressed in muscle repair (i.e., *Fos*, *Egr1*, and *CCN1*). The heatmap shows the top 100 significant genes in six control and six PBMT-treated TA tissue. Upregulated genes are shown in red and downregulated genes in blue. (**B**) Volcano plot representing differential genes expressed in PBMT. *Fos* was significantly upregulated by 2-fold and *Egr1* by a greater than 2-fold expression. (**C**) Upregulated biological processes, including ~40 genes of specific interest in muscle tissue development. (**D**) Upregulated molecular processes, including ~25 genes of specific interest in transcription activity and gene regulation.

Since the mechanism of PBMT is still unclear, we performed RNA-Seq analysis to obtain a better understanding of the genes activated and regulated through PBMT. In addition, we wanted to assess whether PBMT has the ability to influence gene transcription in sedentary muscle tissue. A heatmap representing the 100 most significant genes in regards to sedentary control and PBMT is shown (Figure 1A). In both groups, the gene expression response is consistent across specific genes. PBMT-treated TA muscle displayed ~586 upregulated and ~802 downregulated genes. The volcano plot represents the fold change with significance. Several genes involved in tissue development are of interest, e.g., *CCN1, Egr1,* and *Fos* expression, which play key roles in tendon and muscle regeneration [10,11,12] (Figure 1A,B). One gene of interest that was downregulated was *Nfil3*, also known as *E4BP4*, with roles in regulating the circadian rhythm, immune system, and metabolism [13,14]. In the gene enrichment analysis, the greatest number of upregulated genes, ~40, were involved in muscle tissue development and organ growth (Figure 1C). Most of the upregulated genes were involved in molecular processes regulating transcriptional activity (Figure 1D).

**Figure 2:**
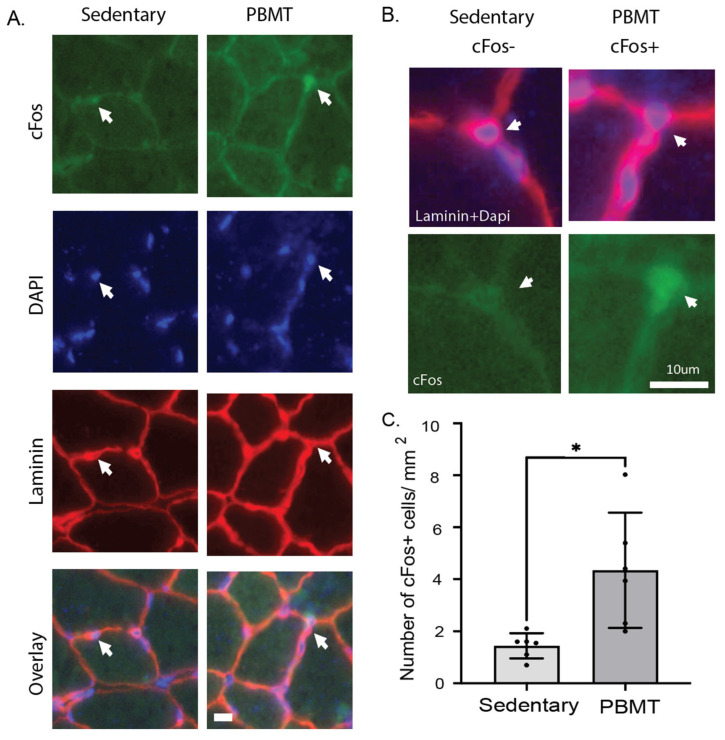
cFOS is upregulated in cells surrounding myofiber in PBMT-treated muscle. (**A**) Muscle tissue sections were stained for laminin (red), nuclei (blue), and cFOS (green). Non-myofiber cells were identified by a laminin border surrounding a nucleus. cFos+ cells are indicated by a white arrow. (**B**) A cFos- cell is represented for sedentary tissue with a low signal for cFos immunostaining, and a cFos+ cell with an increased signal is shown for PBMT-treated tissue. (**C**) The number of cFos-positive cells was counted for the entire TA 10 µm cross section. *N* = 6; * *p*-value < 0.05. Scale bar represents 10 μm.

Muscle regeneration is triggered by satellite and supporting cells present in the tissue. Normally, satellite cells remain in a quiescent state and reside beneath the basal lamina of the muscle fiber. Interstitial cells play a role in supporting satellite cell function [15] and reside close to the muscle fiber, are above the basal lamina, and are surrounded by their own laminin border (Figure 2A,B). Upon encountering external stimuli, they activate and induce *Fos* [16], a gene that is triggered early and is involved in the muscle differentiation process to express cFos protein [17]. To validate the transcriptomic results, immunofluorescence analysis of cFOS and laminin in TA muscle was performed to assess whether cFOS expression occurred in myofiber or other cell types. The number of cFos+ cells in cross sections of the TA muscle of sedentary and PBMT-treated tissue was significantly increased in cells surrounding the myofiber. PBMT-treated muscle showed a 2.8-fold increase in the number of cFos+ cells, i.e., 4.3 ± 2, whereas sedentary muscle exhibited 1.4 ± 0.4 cFos+ cells (Figure 2C).

### 2.2. PBMT Treatment Enhances Endurance Performance

**Figure 3:**
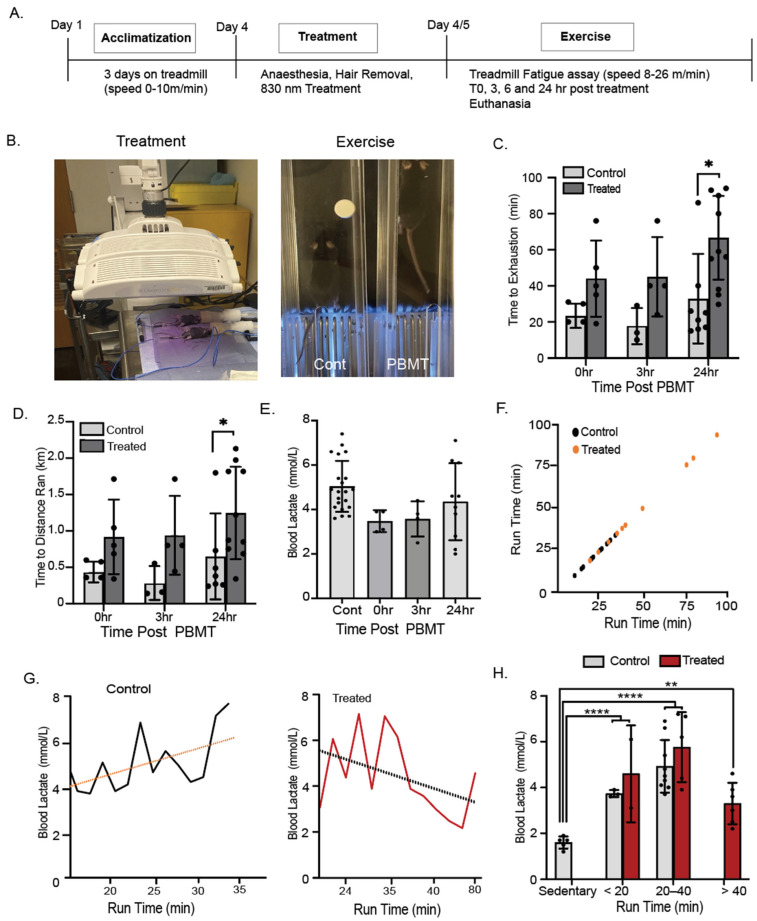
The 830 nm-treated mice showed increased muscle performance. (**A**) Schematic of study timeline. Briefly, following an acclimatization period, mice were treated with 830 nm irradiation on Day 4. A treadmill fatigue assay was performed at time points 0, 3, and 24 h post-PBMT treatment. (**B**) The LED setup used for treatment. Mice were anesthetized, and the hair from their lower limbs was removed by shaving/hair removal cream. Mice were treated with the OmniLux panels (Omnilux Medical, Napa, CA, USA) a 25 mW/cm^2^ for 20 min (30 J/cm^2^). At the designated time points, mice underwent a treadmill fatigue test in which they ran until exhaustion. A representative image of mice running on a treadmill is shown, in which the control mouse ran lower in the lane and was closer to the shock grid when compared with the behavior of the treated mouse. Immediately after capturing this image, the control mouse was removed from the lane, and the treated mouse was allowed to run until it was exhausted. (**C**) At all tested time points, treated mice ran for longer, on average about 1 h, whereas the controls ran about 30 min. (**D**) PBMT-treated mice ran three times the distance of that of the controls. (**E**) Lactate levels in blood were immediately tested after removal of the mouse from the treadmill. Even though the treated mice ran twice as long, the lactate levels were reduced for 0 and 3 h and were similar to those of the control mice for 24 h post-treatment. (**F**) Treated mice were the only group to run longer than 35 min. (**G**) Lactate levels in blood increase with run time in controls, whereas they decline with PBMT treatment. (**H**) Mice were grouped according to run times of <20 min, 20–40 min, and >40 min. Blood lactate was significantly higher for all running groups when compared to the levels for sedentary mice. *N* = 6–10 per group; * *p*-value < 0.05, ** *p*-value < 0.01, **** *p*-value < 0.0001.

**Figure A1:**
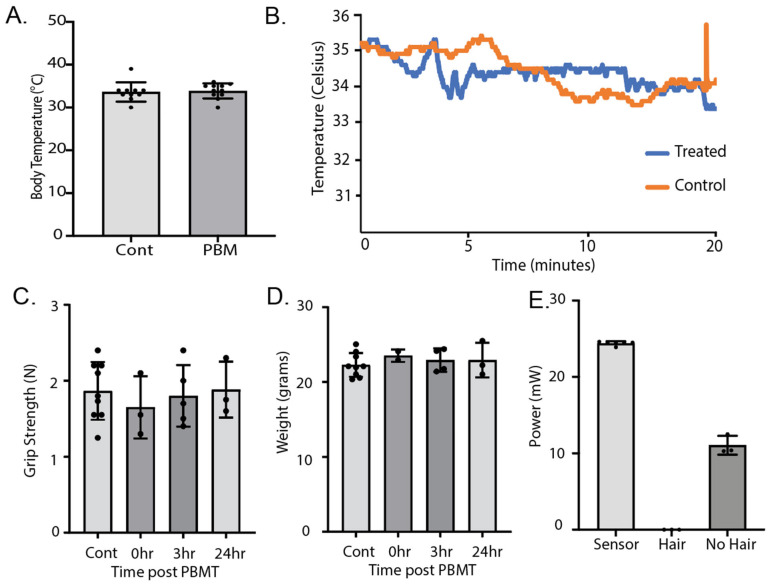
Increase in performance not due to mouse differences or transmission of power through other body parts. (**A**) Core body temperature (which was monitored and maintained as described in the Section 4) at the end of PBMT treatment was similar for all experimental groups. (**B**) Real-time measurement of body temperature during the 20 min treatment process. (**C**) To measure whole body strength, grip strength was measured, with no change between control and treated individuals. (**D**) Body weight of control and treated mice was similar. (**E**) To measure power transmission through skin, with or without hair, ex vivo skin patches from mice were collected after euthanasia. The power measured before placement of skin on the sensor was 25 mW. Skin with hair showed 0 mW detected for three ex vivo skin + hair patches. Hair was then removed with Nair, and the skin was placed on the sensor, detecting ~10 mW of power.

Next, we wanted to investigate the effect of PBMT on exercised muscle to measure its influence on muscle performance. The experimental timeline and PBMT device is represented in Figure 3A,B. Animals were tested for time to exhaustion on the treadmill at three post-PBMT time points: immediately (0 h), 3 h, and 24 h after recovery from anesthesia. At 24 h post-PBMT, the controls ran for 33 ± 25 min, whereas the PBMT-treated individuals ran for 62 ± 25 min, with a significant 2-fold increase in performance (Figure 3B,C). At 24 h post-PBMT treatment, the treated mice ran significantly greater distances (Figure 3D). At 24 h post PBMT, the blood lactate levels were not significantly elevated (Figure 3E); however, mice exhibited varying run durations, and there was a trend of lactate decline with an increasing run time (Figure 3F,G). The run times were categorized as <20 min, 20–40 min, and >40 min. All exercised mice showed a significant increase in blood lactate when compared to that of sedentary mice, and the controls displayed increasing lactate levels with longer run times. There was no difference between the exercised controls and PBMT individuals in regards to blood lactate. (Figure 3H). There was no difference in core body temperature, body weight, or grip strength between the groups (Figure A1).

### 2.3. PBMT Treatment Upregulated Developmental Genes and Attenuated Injury in Fatigued Muscle

**Figure 4:**
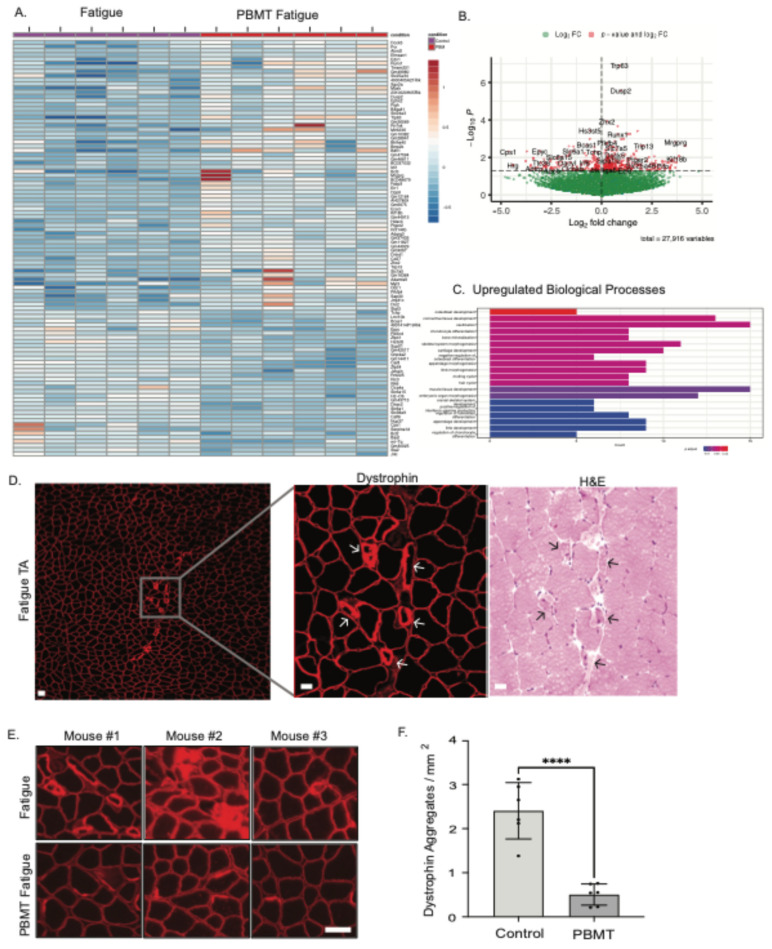
Upregulation of tissue-development genes and attenuation of injury in PBMT fatigued TA. (**A**) Treated and control mice ran on the treadmill until exhaustion, for variable run durations, starting 24 h after PBMT treatment. TA tissue was collected after the fatigue run, and samples were sequenced for RNA. (**B**) Volcano plot depicting top genes, *Trp63*, *Dusp2,* and *Mrgprg*, that are upregulated in acute exercise, involving pain and nerve excitability. (**C**) Biological processes involved in tissue morphogenesis that were upregulated in PBMT-treated TA muscle. Upregulation of ~15 genes in ossification and ~15 genes in muscle tissue development. (**D**) TA fatigued muscle was isolated for histology. Muscle fiber structure was immunostained for dystrophin, a structural protein integral for fiber morphology. Representative dystrophin and H&E staining of consecutive slides. Arrows indicate the same fiber. (**E**) Fatigued muscle fibers showed mild disruption of fiber structure, as seen by dystrophin aggregates, indicating injury which was absent in PBMT-treated muscle. Three representative mice are shown. (**F**) Dystrophin aggregates were counted in the entire TA tissue section for fatigued (control) and PBMT fatigued mice. *N* = 6 mice per group; **** *p*-value < 0.0001. Scale bar represents 50 μm.

The significant effects of 830 nm infrared light on muscle performance prompted us to again assess the effects of PBMT on the molecular level. Thus, 24 h post-PBMT treatment and control mice were euthanized after running on the treadmill; the left TA muscle was dissected for RNA sequencing, and the contralateral limb’s TA and soleus were dissected for microscopy. A heatmap representing the 100 most significant genes in control and PBMT-treated mice is shown (Figure 4A). In controls, the gene expression response is consistent across specific genes; however, there is a larger variability with PBMT treatment. A subset of genes is upregulated in specific mice, with no apparent trend. This may be due to the large variability in run times for mice, both in the control and PBMT groups. A total of 156 genes were downregulated, and 302 genes were upregulated with PBMT treatment. Genes in the top right quadrant of the volcano plot (Figure 4B) are upregulated, with the highest fold change and the greatest significance. The expression of two genes of interest, Trp63 and Dusp2, is increased in the PBMT-treated mice that ran longer. Dusp2 plays a strong role in skeletal muscle adaptation to acute exercise [18], and Trp63 is involved in epithelial development and limb formation [19]. In the gene enrichment analysis, genes were clustered into biological processes (Figure 4C). The most significant upregulation occurred in osteoblast development, indicative of increased muscle and bone crosstalk with PBMT. The greatest number of genes (~15) were involved in connective tissue and muscle tissue development, suggesting tissue adaptation and muscle repair after exercise with PBMT. To assess muscle damage due to exercise, we stained TA tissue with dystrophin, a protein associated with the structural integrity of the muscle fiber membrane during contraction (Figure 4D). In controls that underwent fatigue, the dystrophin is disorganized or aggregated, whereas with PBMT treatment, muscle fiber structure was retained (Figure 4D,E), and the number of dystrophin aggregates was reduced with PBMT treatment (Figure 4F).

**Figure 5:**
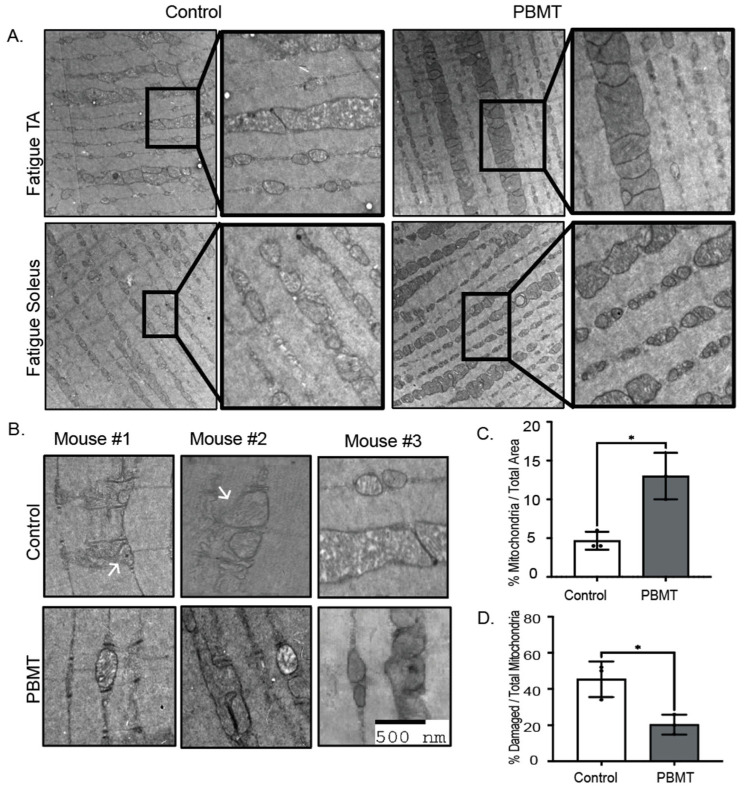
Mitochondrial morphology in fatigued and PBMT fatigued TA. (**A**) After the mice underwent a fatigue run, 24 h post-PBMT treatment, they were euthanized, and TA and soleus tissue was collected for transmission electron microscopy. Longitudinal sections were imaged from three fatigued and three PBMT fatigued mice for each muscle group. Zoomed in panels focus on mitochondrial morphology in control and PBMT groups. (**B**) Higher magnifications of TA muscle are represented to visualize mitochondrial structure. Mitochondria in fatigued mice displayed membrane rupturing (white arrows), and mitochondrial morphology was also swollen where white spacing was present between the mitochondrial cristae. Mitochondria in PBMT-treated mice showed fewer swollen mitochondria, with an abundance of electron-dense mitochondria and with a more fused elongated morphology. (**C**) Quantification of mitochondrial areas in the TEM images obtained from control and PBMT fatigued individuals. Mitochondria were outlined using ImageJ (https://imagej.net/ij/), and total mitochondrial area per image was measured. Images were captured at 2750× magnification. (**D**) Number of damaged mitochondria (as shown in (**B**)) were counted using ImageJ for control and PBMT fatigued TA. *N* = 3 mice per group; *N* = 10–15 muscle fiber per mouse. * *p*-value < 0.05. Scale bar denoted 500 nm.

At the organelle level, we observed altered mitochondrial morphology in control versus PBMT fatigued TA and soleus muscle. In both muscle groups, controls displayed fewer electron-dense mitochondria than did PBMT individuals (Figure 5A). At higher magnification, controls exhibited a higher proportion of mitochondria that were swollen and displayed disrupted membranes (Figure 5B). Total mitochondria and abnormal mitochondrial morphology were quantified from TEM micrographs. Mitochondrial content was higher and less damaged, i.e., 20% in PBMT fatigued TA, whereas in controls, 45% of the total mitochondria appeared damaged (Figure 5C,D). Mitochondrial morphology was protected ~2.2 fold with PBMT, suggesting efficient energy metabolism and recovery from exercise-induced damage.

## 3. Discussion

Our study suggests that 830 nm PBMT pre-treatment can significantly improve endurance performance on a treadmill, possibly through the activation of cells involved in muscle repair. To our knowledge, this is the only in vivo study in healthy mice focused on treadmill-induced fatigue.

Skeletal muscle can adapt rapidly to acute exercise [18], and under high intensity, muscle is damaged due to mechanical, metabolic, and cellular stress. Depending on the level of exercise intensity, fiber structure can become damaged, contributing to pain and soreness [20], and the mitochondria becomes damaged and dysfunctional [21]. In this study, PBMT enhanced treadmill performance 2-fold, and fatigued muscle showed a ~5-fold reduction in dystrophin aggregates and a ~2-fold decline in damaged mitochondria. The increase in performance and mitigation of injury from intense exercise suggests that PBMT increased the regenerative potential of the tissue. In the literature, the application of PBMT is shown to reduce pain after an intense exercise session and to lower the amount of muscle damage molecules in the blood [22]. Similarly, studies have reported the reduction of inflammation in injured areas with the use of PBMT, resulting in improved muscle phenotypes [23].

In uninjured healthy muscles, satellite cells are in a quiescent state with the suppression of cell division. In situations of trauma or injury to the muscle, cells escape the quiescent signaling and begin to proliferate when activated. Interstitial cells play a role in supporting satellite cell differentiation, proliferation, and function. They provide myogenic factors to the satellite cells and are essential for tissue regeneration [15]. cFos is a transcription factor belonging to the activator protein AP-1 family, whose members regulate cell growth, proliferation, and differentiation [24]. One of the earliest events in muscle repair is the expression of *Fos* in satellite activation [25] and its supporting cells [16]. *Fos* expression is necessary for satellite cells to escape quiescence, proliferate, and subsequently activate the regenerative response of muscle. AP-1 associated factors, e.g., early growth response Egrl/2, are also expressed in activated satellite cells [25]. In this study, we see the expression of cFOS in cells surrounding the myofiber, suggesting an increase in the treated muscles’ potential for repair through interstitial and/or satellite cells. The exact cell type displaying cFos expression should be identified through specific cell markers in future studies. PBMT has been shown to activate and proliferate muscle satellite cells in in vivo [5] and in vitro studies [26] of muscle atrophy. Previous literature and our study elucidate the potential impact of photobiomodulation on satellite cells in skeletal muscle.

In our RNA-Seq results, the most significant biological processes upregulated with PBMT were genes involved in muscle and bone signaling. Both skeletal muscle and bone share mesodermal origins and exhibit similar transcriptional signaling programs [27]. During muscle regeneration, genes involved in osteogenesis are activated and may play a role in extracellular matrix remodeling, muscle regeneration, and development. We detected the most significant upregulation in osteoblast development genes, suggesting an increase in muscle regeneration. Several of the enriched pathways are broad (e.g., connective tissue development, skeletal muscle, and organ morphogenesis). These findings are exploratory and require future validation regarding their biological importance in skeletal muscle. Several transcription factors, i.e., Trp63, Sox9, and Smad3, were upregulated with PBMT. These transcription factors play an important role in organ development and regeneration. A homolog of p53, Trp63 is upregulated upon cellular stress and is a transcriptional activator of pathways regulating muscle growth [28]. Sox9, categorized to the biological process of muscle tissue development, affects the development of a variety of tissue, e.g., bone, heart, and lung [29,30]. Smad3 is also involved in regulating gene activity and cell proliferation. An increase in Smad3 signaling results in satellite cell activation and muscle growth and differentiation [31]. These transcription factors, along with several others, suggest that PBMT activated genes involved in tissue regeneration in fatigued muscle.

The expression of approximately 69 genes was similar between PBMT-treated sedentary and exercised muscles. Of particular interest were genes involved in satellite cell activation, (e.g., BMAL1, Smad3, and Dusp4) that were significantly upregulated. BMAL1 is essential for muscle satellite cell proliferation, differentiation, and repair [32]. Smad3 is involved in satellite cell activation [31], and Dusp4 modulates proliferation [33]. Although not significant, cFos expression was increased by 40% in PBMT exercised muscles. The genetic response to PBMT in sedentary and exercised muscle suggest that PBMT treatment triggered muscle repair pathways. Similarly, in 2020, Macedo et al. conducted a study in rats that underwent chronic resistance training and observed that photobiomodulation, when applied prior to the training sessions, increased muscle performance and upregulated genes involved in muscle growth and energy metabolism [7,8].

The variations we observe via transcriptomic analysis (e.g., cFos) in sedentary PBMT mice could be due to several factors. The limb placement under the LED could vary, as well as the amount of hair removed from the limbs and the amount of melanin in the epidermis, hindering light penetration or the endogenous levels of chromophore. This would inherently cause individual variability, as seen in the treadmill performance and subsequent transcriptomics of fatigued muscle of the PBMT groups. Although core body temperature was kept constant between the controls and PBMT group, a sham light or heat-only control was not used. Other limitations of this study should be considered, such as the small number of samples, the focus on fast-twitch muscles, and the sex and age of the mice. In our study, one dose of PBMT pre-exercise was performed to evaluate its biological effects. PBMT response may vary, depending on the wavelength, frequency, and whether the therapy was performed pre- or post-exercise, and these effects should be explored further considering the limitations discussed above.

In conclusion, our study suggests that 830 nm PBMT activated regenerative genes and may have potentially targeted cells involved in muscle repair, rejuvenating the tissue. This resulted in enhanced performance and reduced cellular stress and damage that occurs in acute exercise. Future work is required to establish a mechanistic link between PBMT and the biological mechanisms regulating muscle recovery and repair.

## 4. Methods

### 4.1. Animals

All in vivo experiments were performed on C57BL/6J (Strain #000664). male mice, 5–7 weeks of age, purchase from Jackson laboratory, Bar Harbor, ME, USA. All mice were maintained under a 12 h light and dark cycle and had access to water and food ad libitum. All husbandry and experimental procedures were performed in accordance with the Public Health Service Policy on Humane Care and Use of Laboratory Animals and with approval from the Institutional Animal Care and Use Committee of Massachusetts General Hospital.

### 4.2. PBMT Procedure

Prior to the procedure, the mice were anesthetized, and hair from the lower limbs was shaved, followed by application of a depilatory cream (Nair) for ~20 s, after which the cream was thoroughly removed with water-soaked gauze pads. For the PBMT, we used Omnilux Plus LED panels (Omnilux Medical, Napa, CA, USA) with 830 nm LEDs at an irradiance of 25 mW/cm^2^ and a fluence of 30 J/cm^2^ with an exposure time of 20 min. The control mice underwent similar treatment as that used for the PBMT group. The lower limbs were shaved, and the mice were kept under anesthesia for 20 min to account for the anesthesia time for the PBMT groups. During PBMT, the head/eyes were not covered. The head was placed near the edge of the LED panel, where power output is minimal. Maximum light was delivered at the center of the LED panel where the shaved legs were positioned. Additionally, the remaining body of the mouse, including the head/neck area, was covered with the mouse’s black hair, which effectively stopped light from penetrating into the tissue (Figure A1E). The specification of the LED source is listed in Table 1.

Core body temperature was monitored during the treatment for both control and PBMT-treated mice via a rectal temperature probe and digital meter, and a small fan was applied when needed to counteract any heating from the irradiation so that body temperature was maintained at 34 ± 2 °C. Control mice were kept under anesthesia for the same duration (20 min), without irradiation. All mice were allowed to recover from anesthesia for ~20 min before beginning the treadmill test

All mice, control and treated, were weighed immediately after the PBMT treatment to monitor any changes in weight. Grip strength was also measured, as described in Treat NMD protocol SMA_M.2.1.002 [34]. Briefly, the mice were held by the base of the tail and gently lowered onto the grip meter (Columbus Instruments, Columbus, OH, USA) to allow the forepaws to grab the metal grip bar. The mice were gently pulled away from the grip bar by the tail, and the grip reading was recorded when the mouse could no longer grasp the bar. The procedure was repeated three times per mouse, with a 30 s rest period between each measurement. The mean of the readings was used to calculate absolute grip force.

### 4.3. Exercise Protocol

The mice were acclimatized to the treadmill for 3 days. Briefly, they were placed on an unmoving treadmill (Maze Engineers, Skokie, IL, USA) for 1–3 min and then allowed to run at a speed of 10 m/min. To test endurance performance, a treadmill fatigue test was performed after the acclimation period and 0, 3, and 24 h post-PBMT. All PBMT groups comprised different mice to avoid confounding effects from previous treadmill exercise.

The exercise protocols are listed in Table 2.

Briefly, the angle of the treadmill was adjusted to a 15° incline. The mice were placed on an unmoving treadmill. The treadmill speed was increased by 2 m/min at specific times, as indicated in Table 2, for a maximum run time of 120 min. An electric grid shocker at the end of the treadmill was set at a manufacturer-recommended range of 0.8–1.5 mA. Exhaustion was defined as the point at which the mouse spends more than 10 s on the electric grid shocker without attempting to resume running when nudged or stays within the bottom area of the treadmill lane (the fatigue zone) for longer than 10 s. The time until exhaustion was noted for all animals. The mice were euthanized immediately. In each treadmill fatigue test, 1–2 control mice were present on the five-lane treadmill to minimize potential confounding effects from variations between separate treadmill testing sessions.

### 4.4. Blood Lactate

After removal of the mice from the treadmill, they were physically restrained, and the tail vein was nicked to collect a small drop of blood to measure lactate levels using a Lactate Plus Meter (Nova Biomedical, Waltham, MA, USA). After collection, the bleeding was stopped by applying gentle pressure.

### 4.5. Tissue Collection

The mice were euthanized by 5% isoflurane overdose, followed by cervical dislocation. Left limb tibialis anterior (TA) tissue was collected for RNA extraction and was stored in Allprotect reagent at −80 °C. Right limb TA and soleus tissue was collected for histology and electron microscopy. Briefly, muscle was cut at the mid-belly and placed in a cryomold with O.C.T. and frozen in cold isopentane [35]. Tissue blocks were kept at −80 °C until use. For electron microscopy, muscle tissue was kept in fixative buffer overnight and stored in wash buffer at room temperature until further processing, as detailed below. Tissue dissection was performed 24 h post-PBMT treatment and without PBMT for the controls for sedentary and fatigued mice. For fatigued mice, tissue was isolated as soon as the mice completed the treadmill fatigue test.

### 4.6. Transcriptomics

Whole transcriptomic mRNA sequencing of TA muscle was performed by Admera Health (Admera Health, Plainfield, NJ, USA) for six fatigued controls compared to six fatigued PBMT-treated mice (Project ID: 23263-01-JH mouse) and six sedentary controls compared to six sedentary PBMT-treated mice (Project ID: 23263-02-JH mouse). TA tissue was shipped on dry ice to Admera Health, who processed the samples and performed mRNA extraction, sequencing, and analysis. Total RNA was quantified by Qubit RNA HS Assay (ThermoFisher, Waltham, MA, USA), and RNA integrity was determined using a Bioanalyzer 2100 Eukaryote Total RNA Nano (Agilent Technologies, Santa Clara, CA, USA), with RNA integrity numbers (RINs) of ≥7.7. cDNA libraries were created from the samples using RNA Library Prep Kit for Illumina (New England BioLabs Inc., Ipswich, MA, USA) and sequenced using NovaSeq (Illumina, San Diego, CA, USA). The sample read length configuration was 150 PE for 60 M paired-end reads per sample (30 M in each direction).

### 4.7. Histology

Tissue blocks were cut at 10 µm on the cryostat, and slides were kept at −80 °C until use. Slides were thawed and fixed in formalin, followed by antigen retrieval using 1X sodium citrate buffer (Protein Tech, Rosemont, IL, USA, Cat #PR30001). Samples were then blocked in 2% BSA (Thermofisher, Waltham, MA, USA, Cat #37525) with Mouse on mouse-blocking reagent (Vector Labs, Newark, CA, USA, Cat #MKB-2213-1) for 1 h. For cFos staining, samples were incubated with cFos E8 mouse monoclonal IgG1 (1:100, Santa Cruz Biotechnology, Cat# sc-166940) and Laminin polyclonal Dylight 550 primary Ab (1:200, Invitrogen Cat# PA5-22903) overnight at 4 °C and stained with goat anti-mouse IgG1 AF 488 secondary Ab (1:200, Invitrogen Cat # A21121) the next day. All the antibodies were diluted in 10% goat serum (Abcam, Waltham, MA, USA, Cat #AB7481) and 2% BSA in PBS. After staining, the samples were mounted using Prolong Gold Antifade Mountant with DAPI (Invitrogen, Carlsbad, CA, USA, Cat #P36962) and imaged on a Nanozoomer Digital Pathology scanner. For dystrophin staining, slides were thawed, fixed in acetone, and incubated with blocking solution, followed by overnight staining with Dystrophin monoclonal primary Ab, MANDRA 8B11 (1:100, DSHB Cat# 8B11) at 4 °C. The next day, after PBS washing, the slides were stained with Goat pAb to Ms IgG AF555 secondary Ab (Abcam, Waltham, MA, USA, Cat # AB150114) and mounted in Prolong Gold Antifade Mountant with DAPI (Invitrogen, Carlsbad, CA, USA, Cat #P36962) and imaged using the Nanozoomer (Hamamatsu Photonics, Hamamatsu, Japan).

### 4.8. Transmission Electron Microscopy (TEM)

Samples were processed as described previously [36]. Briefly, samples were washed in 0.1 M sodium cacodylate buffer (wash buffer) several times after fixation. Prior to embedding, tissue was dehydrated in gradient alcohol series. Semithin sections (0.5 μm) were cut for staining with Toluidine Blue. Ultrathin sections (80 nm) were cut for TEM imaging on uncoated 100-mesh copper grids or slots and examined using a transmission electron microscope (Philips, Eindhoven, Netherlands).

Tissue samples were mounted onto the microscope stage, and individual muscle fibers were scanned at a 700× magnification to identify regions with parallel alignment of mitochondria. The magnification was increased to 2750×, and 5–6 high-resolution images of the mitochondria were captured. This process was repeated for 3–4 distinct muscle fibers within the same tissue sample. In total, approximately 12–15 images were obtained at 2750×magnification for each sample. The mitochondrial area was outlined using Fiji [37] for all images, and the average per sample was calculated. Similar analysis was performed for damaged mitochondria. Damaged mitochondria were identified by changes to their ultrastructure, specifically if there was a break in the dark outer membrane and if they were swollen with white spacing present between cristae. The damaged mitochondrial area was normalized to the total mitochondrial area of each image. Image analysis was blinded between two individuals.

### 4.9. Statistics

For RNA sequencing analysis, differential gene expression was performed using De-Seq2 (version 1.4.1). Functional analysis was performed using the ClusterProfiler package with gene sets characterized by biological process, cellular component, molecular function, and Kyoto Encyclopedia of Genes and Genomes (KEGG) status. Significance is reported as an adjusted *p*-value < 0.05. For all time points in Figure 3, *N* = 4–6 mice, and one-way ANOVA with Dunnett’s multiple comparison test was performed. For all other analyses, Student’s *t*-tests were performed in GraphPad Prism version 9.4.1 [Wi or macOS] (GraphPad Software, Boston, MA, USA) when comparing the control to the PBMT-treated group, and *p* < 0.05 was considered statistically significant. All data is reported as mean ± SD.

## Figures and Tables

**Table 1:** Specification of Omnilux Plus LED device modified from Omnilux Medical User Guide, Photo Therapeutics Limited.

Total Output Power	30 +/− 5 W
Output Intensity	55 mW/cm^2^
Output Wavelength	830 +/− 5 nm
Bandwidth	30 nm +/− 5 nm
Lamp Type	LED
Lamp Input Power	500 VA
Input Line Voltage	90 V–250 V
Fuse	T6.3A Ceramic
Input Line Frequency	50/60 Hz +/− 5%
Weight	12 Kg
Dimensions (H × W × D)	370/180/490 mm
Dimensions of LED Head Overall (L × W)	315 × 350 mm
Dimensions of LED Head Active Area (L × W)	150 × 350 mm
Dimensions of single LED Area (L × W)	75 × 70 mm

**Table 2:** Treadmill protocols for acclimatization and treadmill fatigue test.

Acclimatization		Treadmill Fatigue Test	
Time (min)	Speed (m/min)	Time (min)	Speed (m/min)
3	0	1	5
1	2.5	1	8
1	5	1	10
1	7.5	1	12
10	10	1	14
		5	16
		25	18
		15	20
		15	22
		15	24
		40	26

## Data Availability

The datasets generated and/or analyzed during the current study are available in the Gene Expression Omnibus repository, [GEO Accession number: GSE297318]. All data generated or analyzed during this study are included in this published article.

## Abbreviations

The following abbreviations are used in this manuscript:

AP-1	Activator Protein 1
BSA	Bovine Serum Albumin
CCN1	Cellular Communication Network Factor 1
COX	Cytochrome c Oxidase
DAPI	4′,6-diamidino-2-phenylindole
DUSP	Dual Specificity Phosphatase
Egr1	Early Growth Response 1 Factor
cFos	Cellular Fos (Protein of Fos Gene)
GO	Gene Ontology
KEGG	Kyoto Encyclopedia of Genes and Genomes
LED	Light Emitting Diode
MAPK	Mitogen-Activated Protein Kinase
MKP	MAPK Phosphatase
Ms	Mouse (used in antibody naming, e.g., Ms IgG)
NIR	Near-Infrared
O.C.T.	Optimal Cutting Temperature (compound)
PBMT/PBMT	Photobiomodulation (therapy)
PBS	Phosphate Buffered Saline
RIN	RNA Integrity Number
RNA-Seq	RNA Sequencing
TA	Tibialis Anterior (muscle)
TEM	Transmission Electron Microscopy
